# Cefotax-magnetic nanoparticles as an alternative approach to control Methicillin-Resistant *Staphylococcus aureus* (MRSA) from different sources

**DOI:** 10.1038/s41598-021-04160-4

**Published:** 2022-01-12

**Authors:** Manar Bahaa El Din Mohamed, Fatma I. Abo El-Ela, Rehab K. Mahmoud, Ahmed A. Farghali, Shymaa Gamil, Sahar Abdel Aleem Abdel Aziz

**Affiliations:** 1 Department of Hygiene, Zoonoses and Epidemiology, Faculty of Veterinary Medicine, Beni-Suef University, Beni-Suef, 62511 Egypt; 2 Associate professor of Pharmacology, Department of Pharmacology, Faculty of Veterinary Medicine, Beni-Suef University, Beni-Suef, 62511 Egypt; 3 Department of Chemistry, Faculty of Science, Beni-Suef University, Beni-Suef, 62511 Egypt; 4 Department of Materials Science and Nanotechnology, Faculty of Postgraduate Studies for Advanced Sciences, Beni-Suef University, Beni-Suef, Egypt

**Keywords:** Biochemistry, Ecology, Microbiology, Environmental sciences, Medical research

## Abstract

This study aimed to evaluate the efficacy of magnetic nanocomposite of cefotax against MRSA. A total of 190 samples were collected from milk, farm personnel and different environmental components from the dairy farm under the study to isolate *S. aureus*. Cefotax based magnetic nanoparticles was synthetized by the adsorption method and marked using Fourier-transform infrared spectrum (FT-IR), and X-ray diffraction (XRD), then it was characterized using Scanning and Transmission Electron Microscope (SEM and TEM). The obtained results revealed that number of positive samples of *S. aureus* isolation were 63 (33.1%), mainly from feed manger followed by milk machine swabs (60.0 and 53.3%, respectively) at X^2^ = 48.83 and *P* < 0.001. Obtained isolates were identified biochemically and by using molecular assays (PCR), also *mec* A gene responsible for resistance to cefotax was detected. Testing the sensitivity of 63 isolates of *S. aureus* showed variable degree of resistance to different tested antibiotics and significant sensitivity to cefotax based magnetic nanoparticles at *P* < 0.05. It was concluded that dairy environment might act a potential source for transmission of MRSA between human and animal populations. In addition, cefotax based magnetic nanoparticles verified an extreme antimicrobial efficacy against MRSA.

## Introduction

*Staphylococcus* spp. is world-wide pathogen of growing interest that cause several human and animal infections^1,2^. It is a common cause of mastitis and a primary cause of antibiotic resistance in dairy farms^3^. The overuse and /or abuse of antibiotics in dairy and other food production animals consider a major basis of antimicrobial resistance emergence among zoonotic pathogens^4^.

Methicillin resistant *Staphylococcus aureus* (MRSA) has been emerged as a major public health threat owing to increased morbidity and mortality rates in both human and animal infections^5,6^. MRSA infected animals not only possess risk to farm personnel but also can enter food chain through raw milk^7^. MRSA is known to have the ability of developing resistance to antibiotics which is usually acquired through horizontal gene transfer or mutation^8^. Also, it is noteworthy that the origin of this pathogen is complicated, and it can spread through the livestock environment with unknown actual mechanisms^9^. Worth mentioning that MRSA strains are known to be not only resistant to methicillin but also amoxicillin, penicillin and oxacillin^10^. *S. aureus* resistance to methicillin is primarily due to *mec* A gene expression that codifies the protein PBP2a that has low affinity to methicillin and to all β-lactamases^11^.

In recent years, due to increased antimicrobial resistance, concerns have generated a search for innovative antimicrobial strategies such as antimicrobial nanoparticles^12,13^. Magnetic nanoparticles are one of the current nanotechnology approaches having utmost interest world-wide^14,15^. It was proven that loaded antibiotics on magnetic nanoparticles were of choice in the treatment of infectious diseases caused by resistant bacteria since they enhance their efficacy, minimize their side effects and fasten the recovery of animals^16,17^.

This study aimed to find a new alternative to control MRSA traits isolated from animals, animals' attendants and their environment, by loading cefotax onto magnetic nanoparticles (barium Ferrites) and evaluating their biocidal activity.

## Materials and methods

### Study location and design

This study was performed in a private dairy farm in Beni-Suef locality (coordinates 29° 04' N-31° 05' E) Egypt, between January and July 2019. The farm contained 140 lactating dairy cows grouped according to their milk production into 10 groups (*n* = 14) that kept in a partially sheltered yards with earthy floor. Cows were milked twice a day in an abreast parlor provided with seven milking units and hygienic measures prevailed in the farm were generally fair to moderate. Samples collected during the study were approved by International Animal Care and Use Committee (IACUC) and Institutional Review Board (IRB) of Beni-Suef University. All collected samples were cultured for isolation of *S. aureus*. Polymerase Chain reaction (PCR) was used for molecular identification and then tested for their sensitivity against different 6 antibiotics using disc diffusion method, then resistant isolates to cefotax were subjected to *Mec* A gene detection, finally the resistant isolate were tested for their sensitivity to cefotax–barium ferrite using Minimum Inhibitory Concentration (MIC) and Minimum bactericidal concentration (MBC) techniques.

### Sample collection

Milk samples (*n* = 50), were collected aseptically according to the ethical standards of Institutional Animal Care and Use Committee (IACUC), Ref. No: IORG 0,001,050), Beni-Suef University. Environmental samples including swabs from milk machine, water trough, feeding manager (*n* = 30 each) were collected aseptically. The animal samples were obtained after getting an oral consent from the farm manager and animal owners. The samples were stored on ice to be sent to the lab for further bacteriological examination^18^. Moreover, human samples (hand and nasal swabs) (*n* = 25 each) were gathered from workers in the farm under investigation after taking an oral approval from them and in accordance with Institutional Review Board (IRB), Ref. No: IORG 0,001,050), Beni-Suef University. Some of these workers were apparent healthy while few showed furuncle and carbuncle, swabs were obtained from all workers managing the animals with or without clinical symptoms.

### Isolation and identification of *S. aureus*

**Figure 1:**
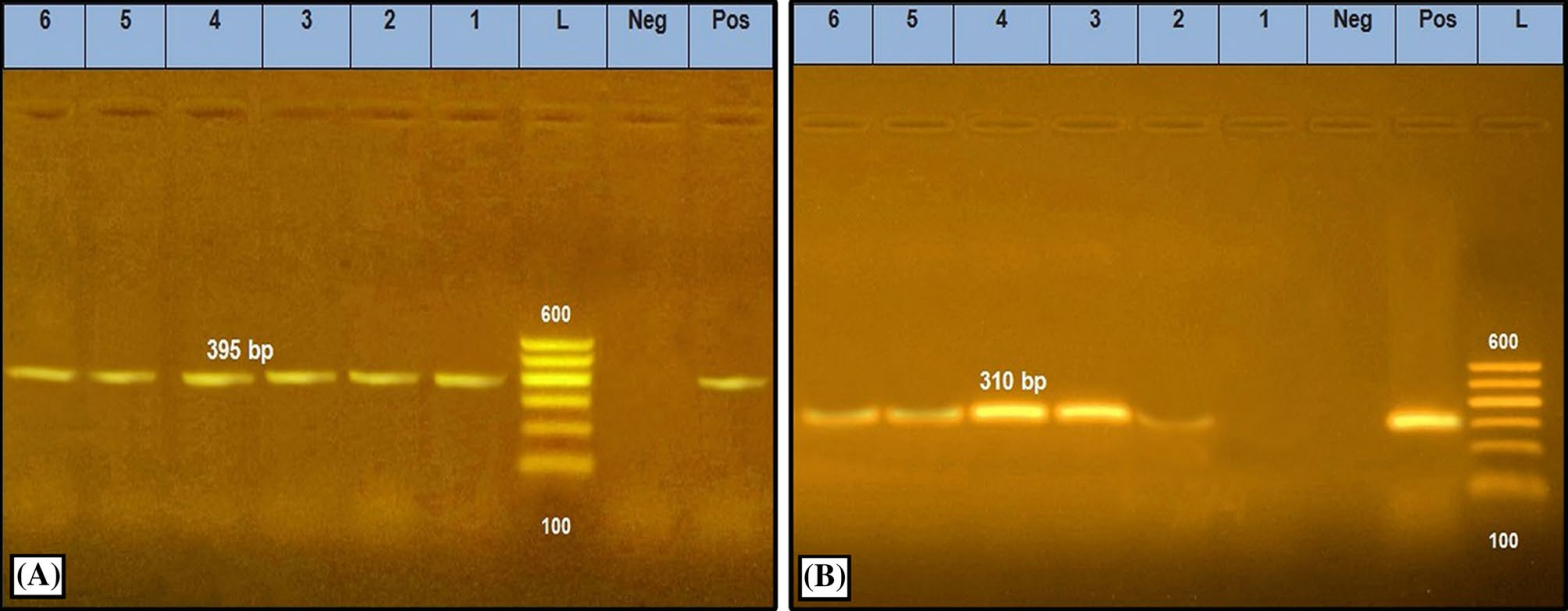
PCR amplification specific for *S. aureus* isolates on *Nuc* (**A**) and *Mec*A (**B**) genes amplified 395 bp, and 310 bp, respectively. Lane (L): 100 bp Ladder ''Marker'', Lanes (1–6): examined samples, Lane Pos: Positive control, Lane Neg: Negative control.

All swab samples (*n* = 140) were pre–enriched on tryptic soy broth (Oxoid, Basingstoke, UK) for 18–24 h at 37 °C then a loopful from each tube showing turbidity was cultivated on the surface of Baird Parker Agar plates (BRA, Oxoid, Basingstoke, UK) and incubated for 24–48 h at 37 °C^19^. One loopful from each milk sample (*n* = 50) was directly inoculated on BRA plates supplemented with egg yolk tellurite and incubated for 24–48 h at 37 °C. Typical colonies on BPA plates were picked and identified by amplification of *S. aureus* specific *Nuc* gene and *Mec*A gene responsible for resistance to cefotax antibiotic^20,21^. Molecular characterization was individually operated for each one primer pair using 40 pmol of each primer, 1 U of platinum Taq DNA polymerase, MgCl2 (Invitrogen), dNTPs and 5µL of target DNA in a total volume of 25µL for each reaction. The thermocycling parameters were summarized as following; an initial denaturation cycle at 94 °C for 5 min then 30 cycles of the subsequent program: 94 °C for 30 s, the annealing temperature was 55 °C for 45 s for each primer. The final extension stage was 72 °C for 7 min. The PCR reaction cycles were performed in an applied biosystem 2720 thermal cycler. The secondary PCR products were separated by electrophoresis in 1.5% agarose gel (Applichem, Germany, GmbH) in 1 × TBE buffer at room temp using gradients of 5 V/cm for 30 min and finally the gel was photographed through UV transilluminator (Alpha Innotech, Biometra) and analyzed through computer software. The molecular characterization was performed in biotechnology unit in the Animal Health Research Institute, Egypt (Table 1 and Fig. 1).

**Table 1:** Sequences of target genes and amplicon sizes specific for *S. aureus* in the current study.

Target gene	Primer sequences	Amplified segment (bp)	References
*Nuc*	ATATGTATGGCAATCGTTTCAAT	395	Gao et al.^20^
	GTAAATGCACTTGCTTCAGGAC		
*MecA*	GTA GAA ATG ACT GAA CGT CCG ATA A	310	McClure et al.^21^
	CCA ATT CCA CAT TGT TTC GGT CTA A

### Antimicrobial susceptibility testing of *S. aureus*

All obtained isolates (n = 63) were tested for susceptibility to six commonly used antibiotics in the veterinary and human practice including: amoxicillin clavulanic acid (25 μg), kanamycin (30 μg), cefepime (25 μg) and oxytetracycline (30 μg) using disc diffusion method. Meanwhile agar dilution method was used to detect MICs of vancomycin (30 μg) and cefotax (30 μg). The bacterial sensitivity was performed using Mueller–Hinton agar (Oxoid, Basingstoke, UK), the agar plates were inoculated aseptically with bacterial suspension at final concentration of 1 × 10^8^ CFU/mL equivalent to McFarland 0.5 that was assessed by BD PhoenixSpec, Nephelometer Becton Dickinson and Company, Sparks, Maryland, USA). Both disc diffusion and agar dilution methods were performed according to CLSI^22^.

### Preparation of Cefotax adsorption on magnetic NPs (Barium Ferrites)

Cefotax–Barium Ferrite was synthesized by adding a solution of cefotax sodium (one gm of cefotax being dissolved in 50 ml water, and the pH adjusted to pH = 7 using NaOH) to barium ferrites for 24 h at room temp. The precipitate was filtered by means of filter paper (Whatman), washed for several times using double-distilled water, finally dried at 40 °C^23^.

### Characterization of Cefotax magnetic nanoparticles

The crystal structure and crystallinity of materials were characterized by X-ray diffraction (XRD). The vibrations of the materials chemical bonds were examined by Fourier transform infrared spectroscopy (FT-IR, Bruker Vertex 70). The particle sizes and zeta potentials were studied (experimentally optimized S1) by a Malvern instrument (Malvern Instruments Ltd). The method of sample preparation was mentioned in a previous work by Abo El-Ela et al.^23^ as well the morphological shape and average nano size of cefotax–barium Ferrite was characterized by SEM and TEM.

### Evaluation of biocidal activity of Cefotax magnetite nanoparticles

**Figure 2:**
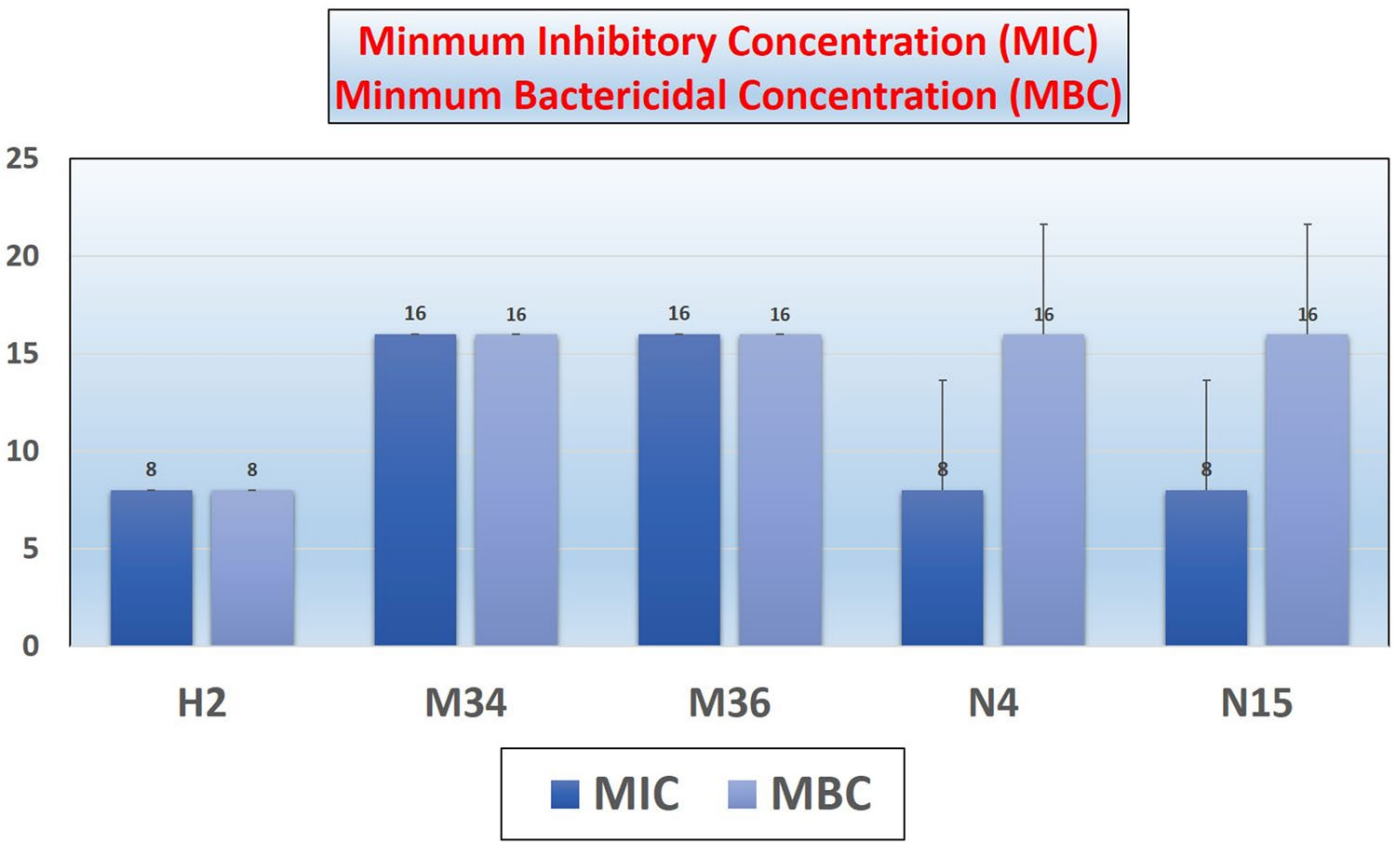
MIC was determined by micro-broth dilution technique and values (Mean) obtained in triplicate for both MIC and MBC.

The antimicrobial activity of the cefotax–barium ferrite was evaluated by Minimum inhibatory concentration (MIC) where the antimicrobial activity of the Cefotax magnetite nanoparticles was evaluated through the broth dilutions in dark conditions. Tubes with no visual turbidity were the endpoint of the MIC, while in minimum bactericidal concentration (MBC) the plates showed no colonies were the endpoint. The antimicrobial activity of cefotaxime-based magnetite nanoparticles had been investigated through the broth standard dilution method; MIC evaluated as the lowest concentration of the nanomaterial or drug visually inhibit the bacterial growth which had been grown on MHA media at 37 °C for 24 h. Cefotax magnetite nanoparticles diluted with Muller Hinton broth for obtaining final concentration of (1000–6.25 µg/ml) in comparison with control positive and negative ones. Bacterial suspension obtained through adding of 5 ml saline to bacterial broth to finally obtain 1 × 10^8^ CFU/ml according to 0.5 McFarland standard tube of the bacteria that previously was sub-cultured on MHA and incubated at 37 °C for 24 h. MIC is the tube of the lowest concentration showed visually complete inhibition of the bacterial growth. Minimal Bactericidal Concentration (MBC), is the tube showed inhibition in the bacterial growth (MIC) besides the before and after 100 µl had been taken from these tubes on MHA plates then incubated at 37 °C for 24 h. Also, the lowest concentration that inhibits 100.0% of the bacterial growth that is observed on the MHA plates after 24 h incubation at 37 °C as demonstrated in Fig. 2. Plates showed no bacterial colonies are considered^23,24^.

## Statistical analysis

The collected data were reported using the Microsoft Excel spreadsheet and prepared for statistical analysis. The frequency of *S. aureus* isolated from different examined samples and biocidal activity of cefotax–barium ferrite against *S. aureus* were calculated using nonparametric tests (Chi-Square Test) by using statistical package for social sciences (SPSS, Inc., version 22.0, Chicago, IL, USA).

## Results and discussion

### The prevalence of *S. aureus* isolation from the different examined samples

Staphylococcal infections represent a public health issue in hospitals and health care settings as well as a major economical and welfare problem in dairy animal farming^25^. The prevalence of *S. aureus* isolation from the farm under the study (Table 2) showed that 63 (33.1%) out of 190 different samples were bacteriologically positive. Moreover, the isolation was mainly obtained from manager swabs followed by milk machine swabs, nasal swabs and hand swabs (60.0, 53.3, 40.0 and 28.0%, respectively), and to a lesser extent in milk samples (24.0%). Meanwhile it was not isolated at any percent from water trough swabs, at *X*^2^ = *48.8*, *P*** < **0.001. These findings spotlight the great role played by the environment as a reservoir of the bacterial infection to the farm workers and later on consumers everywhere also suggested the great role they play in the dissemination of the infection and the running of the pathogen in the nature between animals and humans, where Fluit^25^ proved that the presence of human MRSA isolates in both mastitic milk or infected dairy cattle suggested the role of the environment in transmission of the pathogen between animal and human populations through contaminated utensil, Fessler et al.^26^ also reported that 20% of personnel hanging the poultry in the slaughter were MRSA positive compared to 1.9% from other personnel, while 8% of poultry arriving to the slaughter were MRSA positive that increased to 35% during the day through contamination which suggested the role played by the environment in spreading of the infection, Spohr et al.^27^ also proved environmental pollution with MRSA through detection of MRSA in different farm animals, Antoci et al.^28^ mentioned that prevalence of MRSA was 36 and 61% from humans and bulk milk tank, respectively that might be attributed to low milk hygienic measures. Furthermore, according to Joubert et al.^29^ MRSA in humans, can be maintained and colonized in the mucosa of healthy individuals, especially nasal mucosa and their skin as a potential carrier of the organism that coincide with our results where we were able to isolate the pathogen by (40.0%) from nasal swabs of farm workers; our finding were in harmony with Juhász-Kaszanyitzky et al.^30^, Suelam et al.^31^ and Suranindyah et al.^32^.

**Table 2:** Prevalence of *S. aureus* isolated from animal, human and environmental samples collected throughout the study.

Samples/swabs	No. examined	No. positive	Percentage (%)
**Animal**			
Milk	50	12	24
**Humans**			
Nasal swabs	25	10	40
Hand swabs	25	7	28
**Environment**			
Feed manager swabs	30	18	60
Water	30	–	0
Milk machine swabs	30	16	53.3
Total	190	63	33.1

X^2^ = 48.83.

*P* < 0.001.

### Sensitivity pattern (%) of *S. aureus* traits to the tested antibiotics

The pattern of antibiotic sensitivity of examined isolates (Table 3) showed variable results where all isolates were resistant to the majority of tested antibiotics where all isolates were significantly resistant to amoxicillin clavulanic acid (*P* < 0.001) with variable degree (feed staff, nasal, hand, milk and milk machine, respectively) (61.1, 60.0, 57.1, 41.7 and 37.5% respectively), beside all isolates showed resistance to cefotax including feed staff (77.8%), milk samples (75.5%), milk machine (75.0%), hand swabs (71.4%) and finally nasal swabs (70.0%). Also, most of the obtained isolates from milk machine, nasal swab, feed staff and milk showed resistance to oxytetracycline (68.7, 50.0, 44.4 and 41.7%, respectively). Meanwhile all isolates were significantly sensitive to cefotax magnetic nanocomposite (*P* < 0.05) where the sensitivity by milk machine reached up to (87.7%), milk and feed staff (83.3% each), hand swabs (71.4%), and nasal swabs (60.0%). Moreover, all isolates obtained (milk, feed staff, hand swabs, milk machine and nasal swabs, respectively) were also significantly sensitive to vancomycin at *P* < 0.05 (83.3, 66.6, 57.1, 56.3 and 50.0%, respectively). On the other hand, the recovered isolates showed variable degree of resistance and sensitivity to cefepime and kanamycin but mainly resistance especially among environmental traits. The illustrated results suggested that antibiotic resistant traits of *S. aureus* are no longer exclusive issue but still in the same time pose a high risk due to the zoonotic importance of this pathogen also the possibility of transmission of resistance genes of both disinfectants as well other antibiotics between man and animal populations^33^. The obtained results are to some extent in agreement with Tong et al.^34^ who indicated that drug-resistant strains are growing to represent serious health threats specially MRSA, and Kayvan et al.^35^ who proved that *S. aureus* isolates were resistant to oxacillin (71.15%), cefoxitin (67.31%) and tetracycline (69.81%) and Tenhagen et al.^36^ who mentioned that 100.0% of *S. aureus* were resistant to cefoxitin and penicillin. In contrast Cihalova et al.^37^ found that MRSA were resistant to second-line treatment such as vancomycin and doxycycline. And Jamali et al.^38^ reported lower rates of resistance to oxacillin, penicillin and cefoxitin (13.0, 44.4 and 4.9%, respectively).

**Table 3:** *In -vitro* antibiotic sensitivity pattern of *S. aureus* isolates from animal, human and environmental samples.

Samples/ Swabs	No. tested	Antibiotics used																				
		Amoxicillin clavulanic acid (25 μg)				Kanamycin (30 μg)		Cefotax ) 30 μg)			Vancomycin (30 μg)			Cefepime (25 μg)			Oxytetracycline (30 μg)			Cefotax magnetic nanocomposite (1000–6.25 µg/ml)		
		S	I	R	S	I	R	S	I	R	S	I	R	S	I	R	S	I	R	S	I	R
Animal Milk	12	33.3	25	41.7	25	8.3	66.7	8.3	16.6	75.5	83.3	16.7	0	33.3	41.7	25	25	33.3	41.7	83.3	16.6	0
Human	10	10	30	60	40	30	30	20	10	70	50	30	20	40	40	20	0	50	50	80	0	20
Nasal Hand	7	28.5	14.3	57.1	57.1	28.5	14.3	14.3	14.3	71.4	57.1	28.5	14.3	57.1	42.9	0	42.8	28.5	28.5	85.7	0	14.3
Environmental Feed stuff	18	22.2	16.7	61.1	5.5	44.4	50	11.1	11.1	77.8	77.8	0	22.2	16.7	22.2	61.1	38	16.7	44.4	94.4	5.5	0
Milk machine	16	25	37.5	37.5	18.8	18.8	62.5	12.5	12.5	75	56.3	12.5	31.2	50	12.5	37.5	12.5	18.7	68.7	100	0	0
*P* value			0.000			0.004			0.134			0.035				0.003		0.134		0.003		

*R* resistant, *I* intermediate, *S* sensitive.

### Characterization of barium ferrite nanoparticles and barium ferrite cefotax nanocomposite

**Figure 3:**
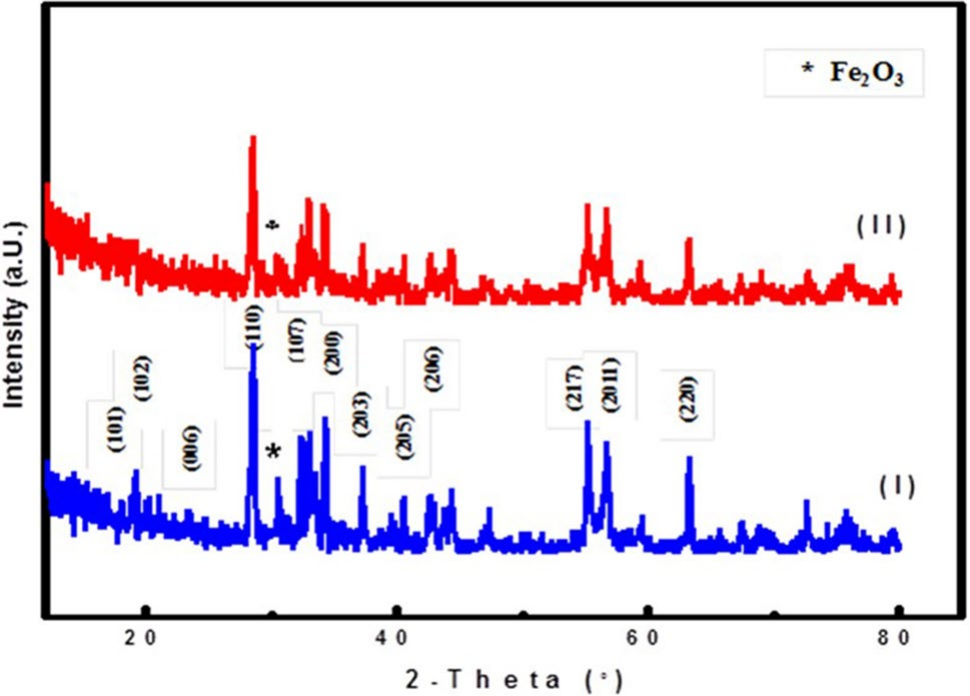
X-ray diffraction patterns of the prepared samples (I) BFO and (II) BFO/Cefotax.

Figure 3 clarifies X-ray diffraction spectroscopy that was used to investigate the crystalline structure of the samples. The X-ray diffraction pattern of barium ferrite showed the specific peak at 2θ at 17*.*79, 18.91, 23.09, 30.39, 32.24, 35.57, 37.17, 40.51, 42.73, 55.19, 56.68 and 63.34 which are corresponding to (101), (102), (006), (110), (107), (200), (203), (205), (206), (217), (2011) and (220) planes of BFO^39^. This result indicates that the barium hexaferrite powder is well crystallized. Regarding barium ferrite/cefotax, the X-ray diffraction pattern (Fig. 3) clearly evidences a slight shifting to a high diffraction angle, a small change in the relative intensity, and a broadening of the diffraction peaks of pure BFO owing to BaF/cefotax formation. This phenomenon is due to the cefotax loading on BFO. And the specific peak of cefotax appeared at 2θ = 33.2, reflections indicating that cefotax is successfully composited with BFO nanoparticles. All the samples exhibit minor peak for hematite phase (marked with asterisks) appeared at 2θ = 31.2 probably because of the preparation method^40,41^. It can be also seen in Fig. 3 that the intensity of the hematite peak shrinks with loading cefotax^42^. Average crystallite sizes of the samples were calculated using Scherer equation and are found to be in range of 23 nm–29 nm.

**Figure 4:**
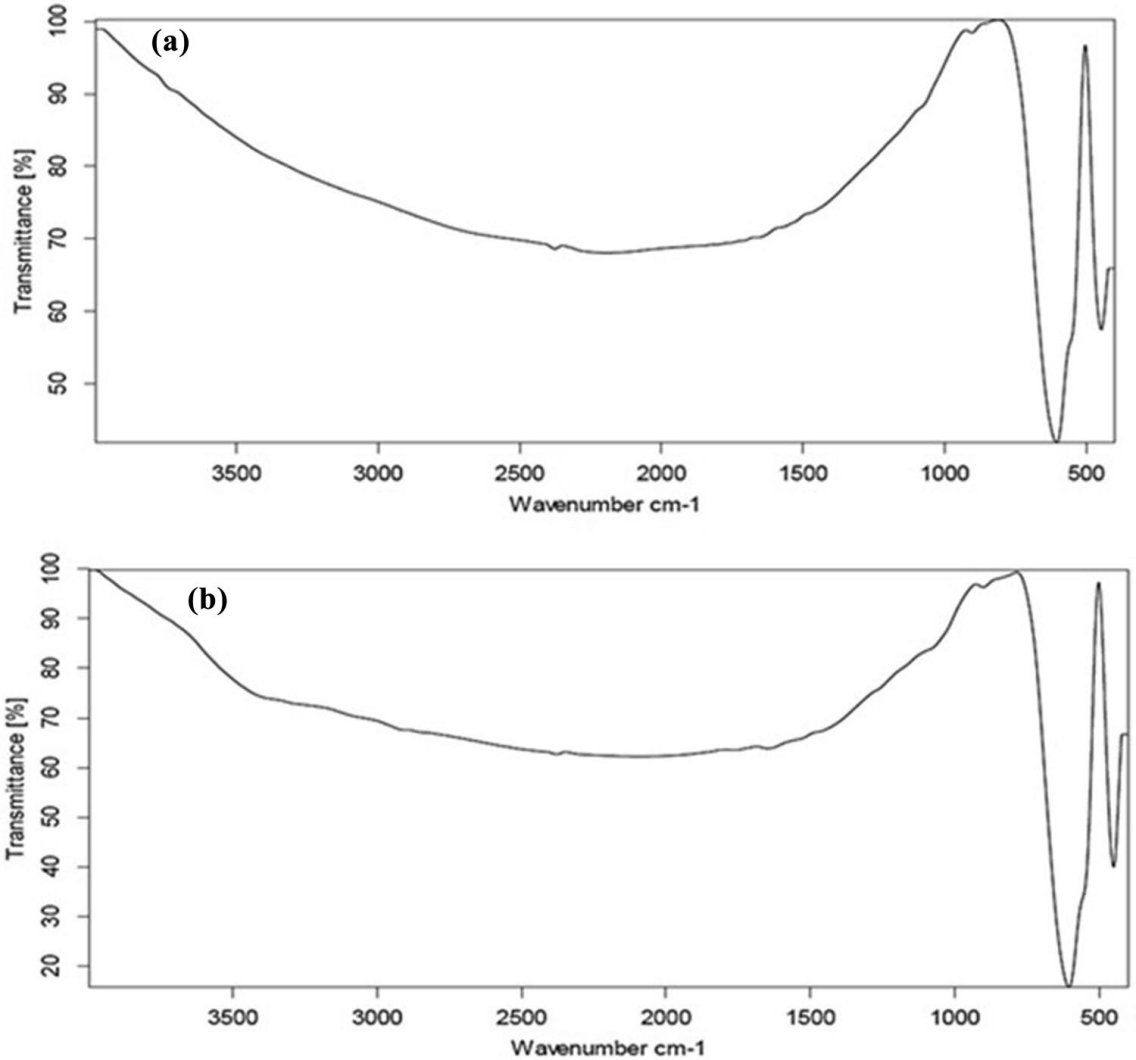
Fourier-transform infrared spectra of the prepared samples (**a**) BFO and (**b**) BFO/Cefotax.

Fourier-transform infrared spectrum was used to identify the functional groups present in the synthesis of samples. The spectrum of pure BFO (Fig. 4a) mainly illustrates characteristic peaks, including 429 cm^−1^ and 583 cm^−1^ corresponded to the lattice vibrations of octahedral and tetrahedral metal ions respectively^43,44^. The intense peak that appeared at 896 cm^−1^ belonged to vibrations from Fe–O or Ba–O^45^. On the other hand, BFO/cefotax, Fig. 4b, spectrum b showed the appearance of important bands of cefotax in the spectra of the BFO /cefotax composite as following: stretching of O–H groups appeared at 3456 cm^−1^, C=O stretching vibration of (COO)^2–^ centered at 1852.76 cm^−1^, N–H stretching is shown around 1569 cm^−1^, the peaks at 1623, 1494.10, 1367.96, 1032, 772, and 610 cm^−1^ are assigned to the presence of O of an amide group, N–H, C=O, C–N, C–O, CH_2_, and C–S groups, respectively^46^.

**Figure 5:**
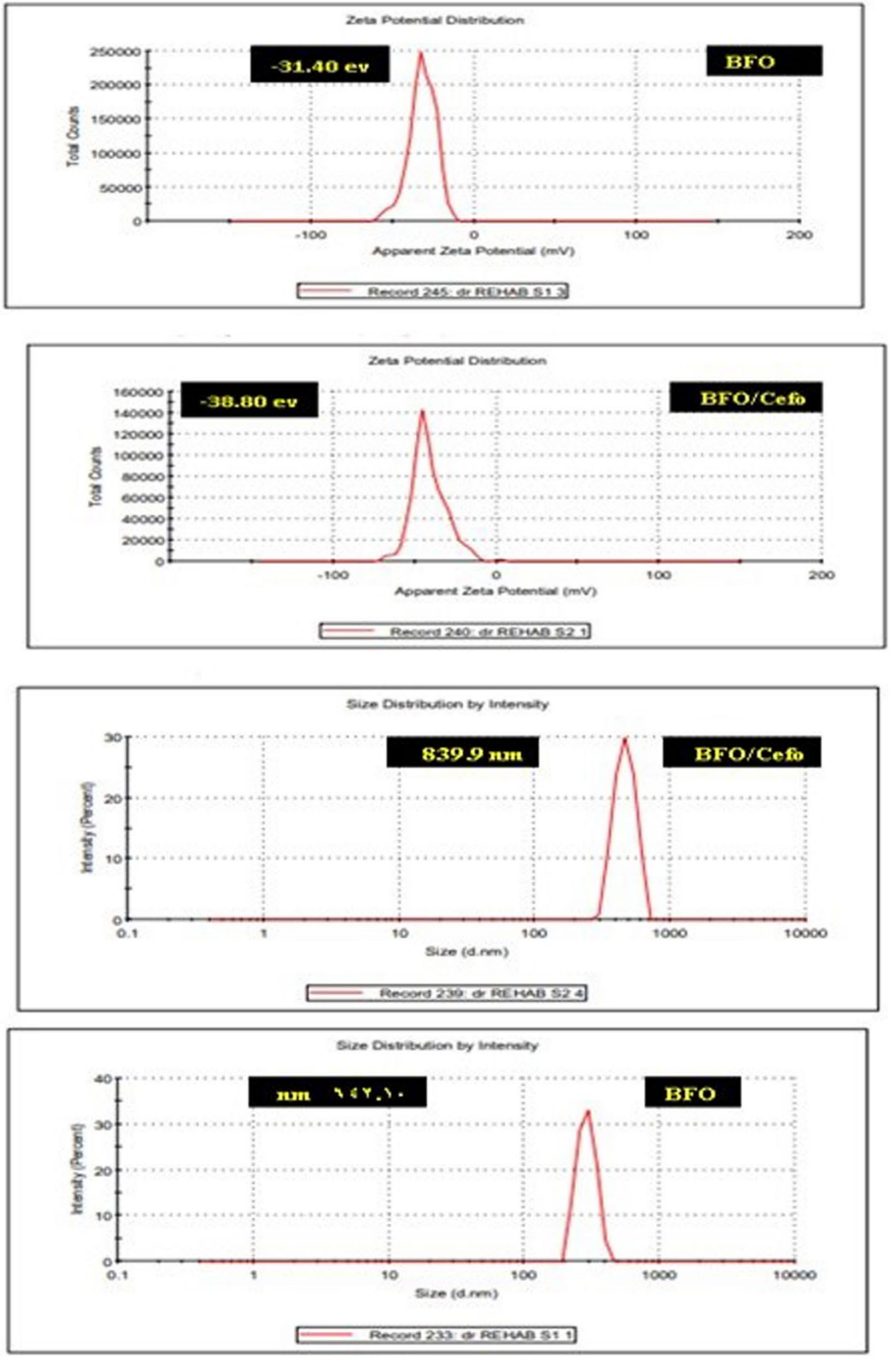
Zeta potential and partial size of the prepared BFO and BFO/Cefotax.

Zeta potential analysis is a measure of the charge attraction/repulsion between particles in solution, suspension stability, and gives us information about the reason of aggregation and dispersion. Figure 5 illustrated the zeta potentials of aqueous dispersions of the BFO and BFO/Cefotax were -31.40 and -38.80 mV, respectively. The results show that the lower zeta potential value of the BFO/Cefotax can be due to the interactions between the inorganic material and organic molecules (cefotax). The decrease is expected to be based on electrostatic considerations. The size distribution intensities of the BFO and BFO/cefotax were 642.10 and 839.9 nm, respectively**.**

**Figure 6:**
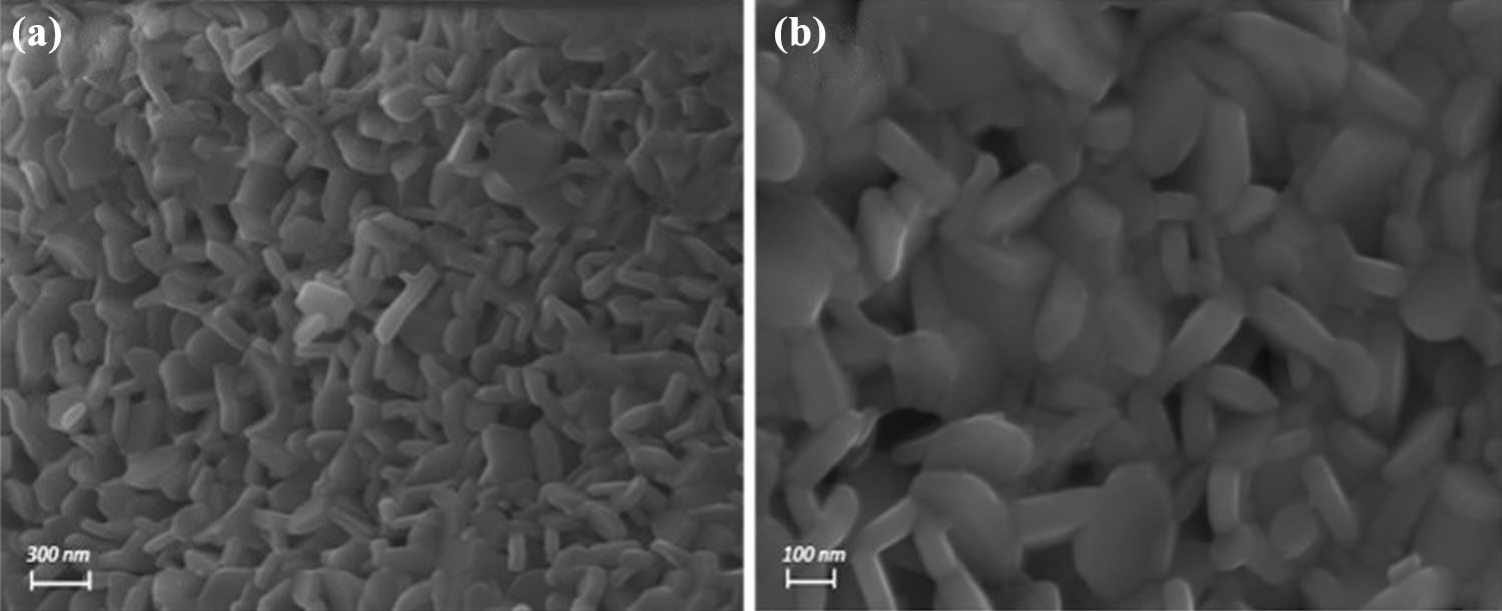
Scanning electron microscopy images of the prepared samples (**a**) BFO and (**b**) BFO/Cefotax.

The surface morphologies of the prepared BFO and BFO loaded with cefotax were studied by SEM, and ideal images of the samples were presented in Fig. 6. As expected, the loading with cefotax resulted in clearly visible microstructure differences of the obtained materials. For BFO formation of ultrafine, irregular-shaped grains (Fig. 6a). The particles are plate like hexagonal /hexagonal in structure. Even though the particles are not uniform hexagonal but mixed of plate like hexagonal/hexagonal. However, for BFO loaded with cefotax, the surface morphology changes to regular grains formed regular hexagonal platelets (Fig. 6b). Therefore, BFO/cefotax composite is expected to be promising catalyst.

**Figure 7:**
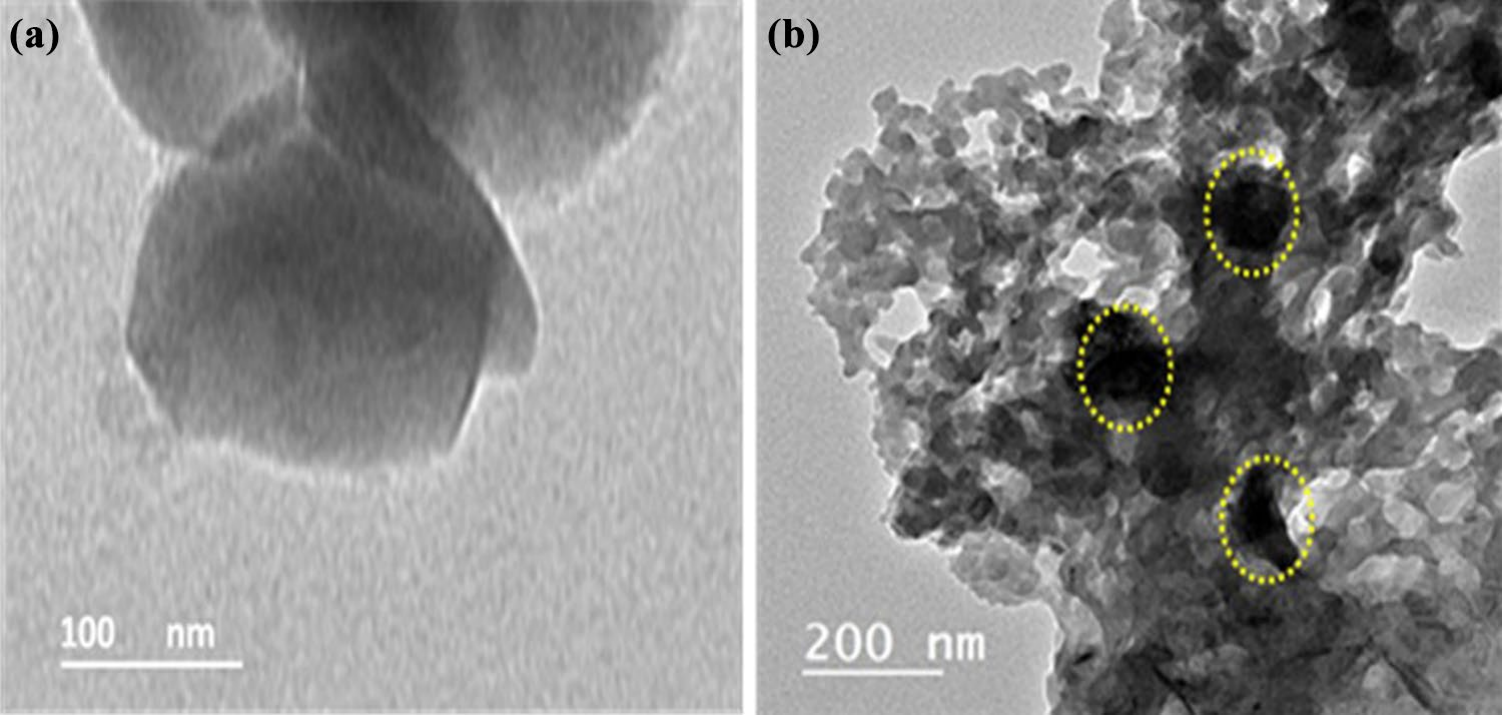
High resolution transmission electron microscopy (HRTEM) images of the prepared samples (**a**) BFO and (**b**) BFO/Cefotax.

Figure 7 shows the high-resolution transmission electron microscopy (HRTEM) micrographs of the BFO and BFO loaded with cefotax. It can be clearly seen that there was an obvious difference between the HRTEM images. Pure BFO consists of layered structure with an irregular shape Fig. 7a. BFO loaded with cefotax image Fig. 7b shows cefotax is distributed on the surface of the barium ferrite nano sheet. The face-to-face assembly of BFO and cefotax optimizes their contact area which is advantageous for controlling MRSA.

At this study a trial for increasing the antibiotic cefotax, one of the methicillin resistant antibiotics, efficiency against MRSA by loading it on magnetic NPs as barium ferrite. The results in the current study showed a good bactericidal activity for the antibiotic loaded on NPs against the resistant bacteria through the MIC, MBC and disc diffusion assay while the magnetic material itself (barium ferrite) showed no antimicrobial activity. The main reason for improvement the antimicrobial activity against the resistant bacteria depends upon mainly their high surface area to volume ratio which gives a chance for the antibiotic to come in contact well with more surface area of the bacterial cell membrane^42^. Magnetic nanoparticles in current nanotechnology approaches is one of the major topics of interest^14,15^. Loading antibiotics on magnetic nanoparticles had an effective strategy in the treatment of infectious diseases caused by resistant microorganisms specially if targeted with external magnetic field as this fasten form the treatment and minimizes the side effects with enhances efficiency^16,17^. Ferrites in particular are non-toxic of low price, biocompatible and their magnetism is very important in different biomedical applications. Also loading of antibiotics on large surface area of magnetite NPs increases the interaction and the penetration of the loaded antibiotics with the bacterial cell surface besides ferrites causes oxidative stress and free radical formation which help in the bactericidal activity of the loaded antibiotics^14,46,47^. In addition, the efficacy of this loading on NPs materials depends mainly upon various factors such as surface modification, natural characteristics of particles, particle composition and the targeted bacterial species. The safety of barium ferrite had been previously studies and indicated the safety of these particles to be used for treatment of infections^15^.

## Conclusions

This study concluded that MRSA was isolated from animals' environment, farm animals' workers and from milk samples and these finding might suggest that the animals' environment may consider the main reservoir for infection both animals and humans, and by that I mean not only the farm animals workers but also human consumers through the consumption of contaminated dairy products. Therefore and owing to the upsurge increase of the pathogen's antimicrobial resistance to the majority of antibiotics classes, an alternative was mandatory to overcome this major problem. Loading antibiotics on magnetic nanoparticles was tested and this study proved that cefotax loaded on BFO had a promising efficacy in controlling MRSA by improving bactericidal power of the drug and increasing its penetration through bacterial cells.

## Supplementary Information

Supplementary Figure 1.

Supplementary Figure 2.
