# Supplementary figures and images for: Cefotax-magnetic nanoparticles as an alternative approach to control Methicillin-Resistant Staphylococcus aureus (MRSA) from different sources
Source: Sci Rep. 2022 Jan 12;12:624. doi: 10.1038/s41598-021-04160-4 (PMC8755787; doi:10.1038/s41598-021-04160-4)

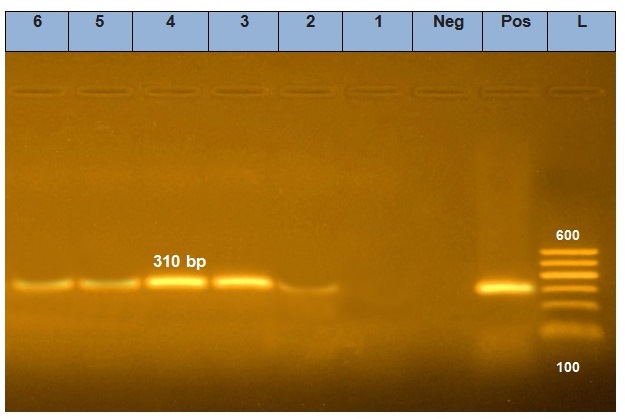

Supplement: Supplementary file 1 — Supplementary Figure 1. [file 41598_2021_4160_MOESM1_ESM.jpg]

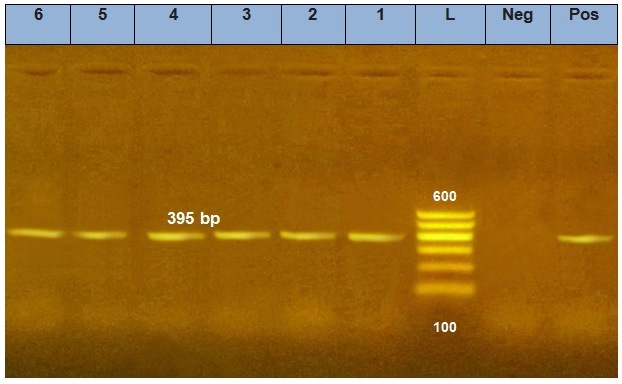

Supplement: Supplementary file 2 — Supplementary Figure 2. [file 41598_2021_4160_MOESM2_ESM.jpg]
